# Influence of β-Stabilizer Element on Microstructure and Mechanical Behavior of Porous Titanium Alloy Synthesized by Liquid Metal Dealloying

**DOI:** 10.3390/ma16165699

**Published:** 2023-08-19

**Authors:** Artem Okulov, Stefan Berger, Ilya Okulov

**Affiliations:** 1 Division of Materials Mechanics, Institute of Materials Research, Helmholtz-Zentrum Hereon, 21502 Geesthacht, Germany; stefan.berger@hereon.de; 2 Department of Particles and Process Engineering, University of Bremen, Badgasteiner Str. 1, 28359 Bremen, Germany; i.okulov@iwt.uni-bremen.de; 3 Leibniz Institute for Materials Engineering—IWT, Badgasteiner Str. 3, 28359 Bremen, Germany

**Keywords:** liquid metal dealloying, porous material, titanium alloy, biomedical material, mechanical behavior

## Abstract

The metallic implant materials for load-bearing applications typically possess a significantly higher stiffness when compared with that of human bone. In some cases, this stiffness mismatch leads to a stress-shielding effect and eventual loosing of the implant. Porous metallic materials are suitable candidates to overcome this problem. In this study, we synthesized low modulus open porous TiFe alloy by liquid metal dealloying of the precursor Ti_47.5_Fe_2.5_Cu_50_ (at.%) material in liquid Mg. Upon liquid metal dealloying, Cu was selectively dissolved from the precursor, and the remaining Ti and Fe elements were reorganized into a bicontinous porous structure. The synthesized TiFe alloy is composed of α-titanium and β-titanium phases. The average measured ligament size is in the micrometer range. It was found that a higher dealloying temperature leads to a pronounced coarsening of the microstructure. The open porous TiFe alloy possesses a low elastic modulus of about 6.4–6.9 GPa. At the same time, its yield strength value reaches about 185 MPa due to the α + β microstructure. Its attractive mechanical properties for biomedical applications, together with its open porous structure, indicate the potential of porous TiFe alloys to be used as implants.

## 1. Introduction

Among metallic materials applied for biomedical implants, titanium-based materials are one of the most prominent candidates. This is due to the combination of their outstanding characteristics, such as a high strength, low density, high corrosion resistance, complete inertness to the body’s environment, high biocompatibility, low Young’s modulus and high ability to osseointegrate with bone and other tissues [1,2,3,4,5]. Despite the above favorable characteristics of titanium alloys, their stiffness value still exceeds that of human cortical bone [3,6]. In some cases, this discrepancy can lead to the so-called “stress-shielding” effect [3,4,6]. This effect provokes bone resorption and leads to a loosening or even complete rejection of the implant from the bone. The development of low modulus titanium-based materials remains the subject of intensive research [7]. Specifically, porous and composite titanium-based materials are promising candidates [8,9]. The porous titanium alloys can be synthesized by additive manufacturing [10,11,12], sintering [13] and dealloying [14,15,16]. The infiltration of a polymer into porous titanium alloys typically improves their strength values while keeping elastic modulus values low [16,17,18,19]. Different dealloying methods can be used to synthesize porous titanium materials, and liquid metal dealloying is one of the most efficient approaches leading to materials with moderate strength values, which are required for biomedical applications [15,18].

**Figure 1:**
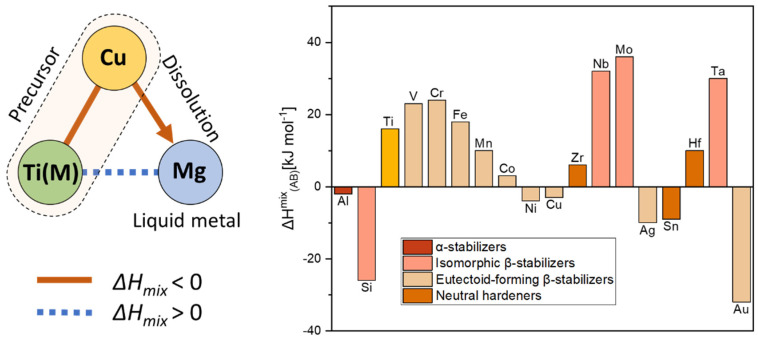
Selection of system for liquid metal dealloying. Relationship of the values of enthalpy of mixing between elements required for the liquid metal dealloying (**right panel**). The values of enthalpy of mixing calculated by Miedema’s model for atomic pairs between Mg and alloying elements for titanium alloys (**left panel**) [35,37].

Liquid metal dealloying is a metallurgical process for the production of porous materials using selective corrosion in a liquid metal [7,14,15,16,18,19,20,21,22,23,24,25,26,27,28,29,30,31,32,33,34]. This method relies on the diffusion of liquid metal into a precursor material, accompanied by the selective dissolution of one or more of its components (Figure 1). Upon dealloying, an interpenetrating-phase or 3D interconnected structure is formed [21,22]. In a following chemical etching step, one of the phases can be chemically removed to obtain a porous structure. The size and morphology of the microstructural features (so called ligaments) of the dealloyed porous materials can be effectively tuned by controlling the dealloying conditions as well as the microstructure of the precursor material [29]. The liquid metal dealloying method enables the synthesis of composite materials [31] possessing physical properties beyond expectations (e.g., based on the rule of mixtures) due to their unique bicontinuous microstructure as well as porous materials ranging from very reactive ones, such as magnesium [32], to advanced ones, such as high-entropy alloys [29,30] as well as titanium alloys.

Several types of porous titanium alloys synthesized by liquid metal dealloying have been reported, namely α (hexagonal close-packed or hcp) [15,19,33], α + β [7] and β (body center cubic or bcc) titanium alloys [34]. The thermodynamic conditions required for the synthesis of porous titanium alloys by liquid metal dealloying are shown below in Figure 1. For example, the precursor alloy can be composed of Ti(M) (M is an alloying element possessing a high positive enthalpy of mixing with Mg) and Cu possessing a high negative enthalpy of mixing with Mg. In this case, the Cu element will be dissolved into liquid Mg upon dealloying. The different phase composition of the dealloyed porous titanium alloys can be achieved by the usage of different types of alloying elements (Figure 1). It is known that depending on the influence of alloying elements on the temperature of the polymorphic transformation α → β of titanium, these are divided into three groups: α-stabilizers, β-stabilizers and neutral hardeners [35] (Figure 1). Alloying elements that increase the temperature of the polymorphic transformation of titanium are called α-stabilizers. These elements are typically divided into two subgroups, namely ones forming solid substitutional solutions (e.g., Al, Ga, In) and ones forming interstitial solutions (e.g., C, N, O). The opposite effect on the polymorphic transformation temperature demonstrates β-stabilizers. There are two types of β-stabilizers: β-isomorphic and β-eutectoid ones. The β-isomorphic stabilizers, such as V, Mo, Nb, and Ta, completely dissolve in the β-phase [7]. The β-eutectoid elements, such as Fe, Mn, Cr, Co, Ni, Cu, and Si, have limited solubility in titanium and form intermetallic phases through eutectoid decomposition. Neutral hardeners, such as Zr, Hf, and Sn, are soluble in both the α- and β-phases. These have a low effect on the temperature of the polymorphic transformation of titanium [35,36].

The study goal is to investigate the influence of a β-stabilizing element on the phase formation, microstructure and mechanical behavior of a liquid metal dealloyed porous alloy. Fe was chosen for this investigation as a strong β-stabilizing element. Two dealloying temperatures were selected, namely below (800 °C) and above (900 °C) the polymorphic transformation temperature. The synthesis and structure–property relationship of the TiFe open porous alloy synthesized by dealloying of the Ti_47.5_Fe_2.5_Cu_50_ (at.%) precursor alloy in liquid magnesium are discussed in detail.

## 2. Materials and Methods

Rods of 1 mm diameter were fabricated from the precursor Ti_47.5_Fe_2.5_Cu_50_ (at.%) alloy by an arc-melting device in combination with a vacuum suction casting machine (Mini Arc Melter MAM-1, Edmund Bühler, Bodelshausen, Germany) under Ar atmosphere. The precursor alloy for the rods was prepared from the pure metals, namely titanium granule (99.99%), copper foil 1 mm (99.99%) and iron wire 1 mm (99.99%) supplied by Alfa Aesar GmbH & Co. KG (Karlsruhe, Germany) and Chempur (Piekary Śląskie, Poland). The alloy was remelted at least 20 times to achieve homogeneity. Then, the rods were cut into 2 mm pieces by a wire saw (Model 3032, Well Diamantssäger, Mannheim, Germany), and these pieces were dealloyed in liquid Mg (magnesium granules with a purity of 99.98% supplied by Alfa Aesar GmbH, Karlsruhe, Germany). These 2-mm-long rods were then systematically heated for various times and temperatures together with ~120–140 mg magnesium (Mg) (−12 + 50 mesh) in a glassy carbon crucible under Ar flow using an infrared furnace (IRF 10, Behr, Düsseldorf, Germany). The precursor Ti_47.5_Fe_2.5_Cu_50_ samples were dealloyed using several dealloying parameters, namely 800 °C for 10 min and 900 °C for 5 min. In order to obtain porous samples, the dealloyed rods were chemically etched in 3M HNO_3_ for 24 h to remove the Mg-rich phase. The microstructure and chemical composition of the porous TiFe samples were investigated by scanning electron microscopy (SEM, Supra 55VP, Carl Zeiss AG, Jena, Germany) coupled with energy-dispersive X-ray (EDX) analysis (Bruker, Mannheim, Germany). The reflections in the X-ray diffractograms were identified using X’Pert HighScore Plus Software 3.0.5 (Malvern Panalytical, Malvern, UK). The average sizes of pores and ligaments were measured through the use of ImageJ Software 1.8.0 (National Institutes of Health, Bethesda, MD, USA). The mechanical behavior of the porous samples was probed under compression loading at room temperature (293 K) and with a strain rate of 10^−4^ s^−1^ using a universal testing device (Z010 TN, Zwick-Roell, Ulm, Germany). The compression tests were designed based on the norm ISO 13314:2011 “Compression test for porous and cellular Metals”. The strain was measured by a laser extensometer (LaserXtens, Zwick, Germany).

## 3. Results

**Figure 2:**
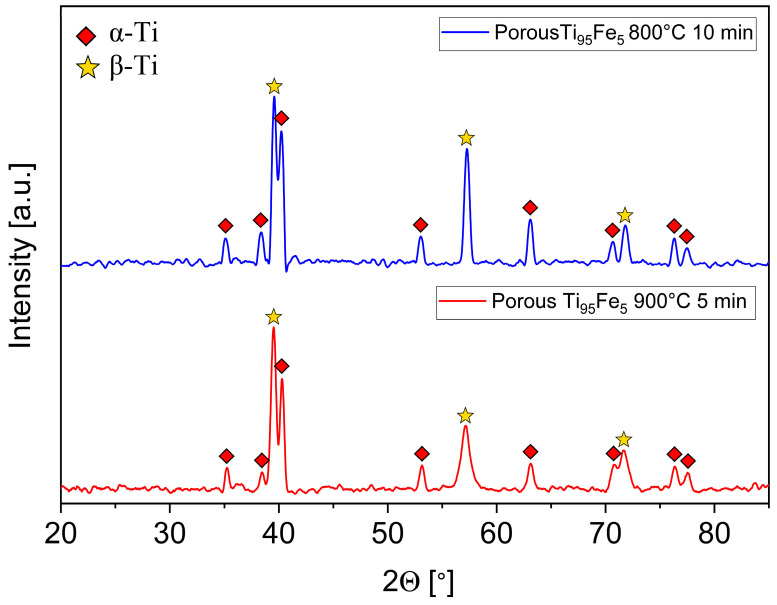
X-ray diffractograms of the porous Ti_95_Fe_5_ (at.%) samples obtained using several dealloying parameters, namely 800 °C for 10 min and 900 °C for 5 min.

The phase composition of the obtained porous TiFe alloys was determined by X-ray diffraction analysis. The resulting XRD diffractograms of the TiFe samples synthesized using two dealloying parameters, namely 800 °C for 10 min and 900 °C for 5 min, are displayed in Figure 2. Both samples are composed of the α- and β-Ti phases. The formation of the α + β structure occurs due to the fact that Fe is a β-stabilizing element for the titanium alloys [35]. According to Ilyin et al. [36], about 3.3–4.3 at.% of Fe is required to stabilize the β phase in Ti-based alloys. In the current TiFe alloy, the expected concentration of Fe is about 5 at.%, which is higher than the critical concentration for the stabilization of the β phase. Moreover, the obtained XRD data are in agreement with [38,39], where the stabilization of β-phases at room temperature is also due to the Fe addition. It is important to note that the width of the α and β peaks increases at a higher dealloying temperature. This might indicate chemical heterogeneities, a finer size or a higher concentration of defects. In the case of the broad β-phase peaks, it seems that this is due to the presence of a nanoscale ω phase in the β grains. The ω-phase peaks are similar to those of the β phase. Further analysis, e.g., transmission electron microscopy, is required to prove this assumption.

The EDX elemental analysis indicates that the chemical composition of the porous TiFe samples is consistent with the expected composition. The chemical compositions of the porous TiFe samples are summarized in Table 1. The insignificant deviation of the measured composition compared with the expected ones is probably due to the surface effects, namely the porosity and roughness. It is highly important to emphasize that biologically harmful Cu was undetected in the porous TiFe samples, although it is present in the precursor alloy used for dealloying. This suggests that the porous titanium alloys consisting of biocompatible elements can be synthesized by LMD process from the Cu-rich precursor alloys.

**Figure 3:**
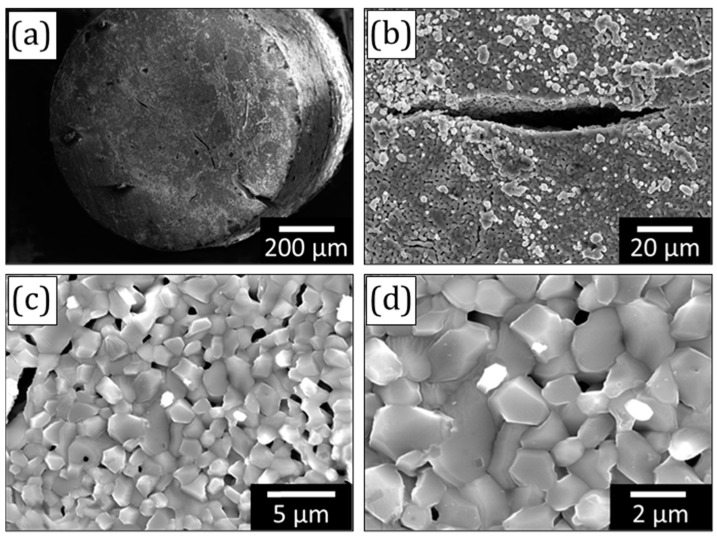
Micrographs of the porous Ti_95_Fe_5_ (at.%) sample obtained by dealloying of the precursor alloy at 800 °C for 10 min: (**a**,**b**) macrocracks on the sample surface, (**c**,**d**) ligament structure.

The microstructure of the porous TiFe sample dealloyed at 800 °C for 10 min is shown in Figure 3. There are several macroscopic cracks detected on the surface of the TiFe samples. The porous structure depicted in Figure 3c,d is similar to that observed for the sintered samples. The average pore size is 0.57 ± 0.24 μm. The ligaments are faceted and sintered with each other. The average ligament size measured by ImageJ software is about 1.5 ± 0.1 μm.

**Figure 4:**
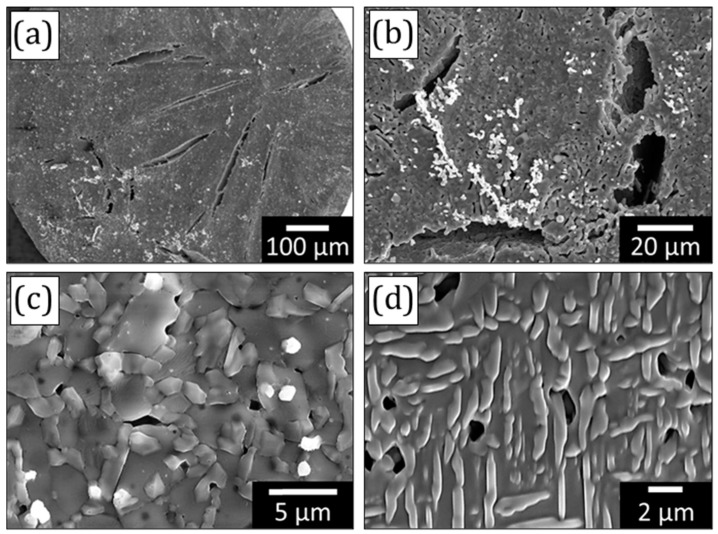
Micrographs of the porous Ti_95_Fe_5_ (at.%) sample obtained by dealloying of the precursor alloy at 900 °C for 5 min: (**a**,**b**) macrocracks on the sample surface; (**c**) ligament structure; (**d**) needle-shape α-phase in the β-phase matrix.

The microstructure of the porous TiFe sample dealloyed at 900 °C for 5 min is shown in Figure 4. Similar to the other sample, there are several macroscopic cracks detected on the surface of the TiFe samples. The porous structure depicted in Figure 4 is similar to that observed for the sintered samples. However, the concentration of pores is lower when compared with the samples dealloyed at 800 °C. The average pore size is 0.55 ± 0.20 μm. The ligaments are faceted and sintered with each other. The average ligament size measured by ImageJ software is about 2.0 ± 0.1 μm. Detailed microstructural analysis reveals a needle-shape phase. This phase has a typical morphology reported for the α and α′ phases in titanium alloys [35].

The observed morphology of ligaments is untypical for porous titanium and its alloys synthesized by liquid metal dealloying. This is likely due to a combined effect of a relatively low concentration of Cu in the precursor and Fe as an alloying element. It is known that the addition of Fe enhances the sintering kinetics of titanium alloys because the mobility of titanium atoms is accelerated by the rapid diffusion of Fe [40]. A higher concentration of Cu in the precursor typically leads to a larger pore spacing [16]. The macrocracks are a typical phenomenon for dealloying materials. In the case of liquid metal dealloying, such cracks are due to inhomogeneous dealloying along grain boundaries.

**Figure 5:**
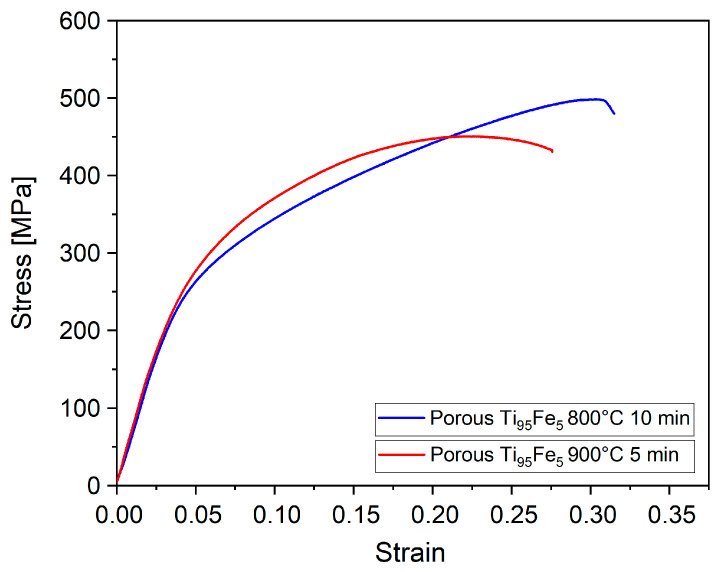
The compression stress–strain curves of the porous Ti_95_Fe_5_ (at.%) samples at room temperature.

The mechanical behavior of the porous samples was probed under compression loading. The stress–strain curves are presented in Figure 5. It can be seen that the mechanical behavior of samples dealloyed under different conditions is very similar. The values of the yield strength and elastic modulus are summarized in Table 2. The yield strength values for both samples reach about 180 MPa. The elastic modulus values are about 6–7 GPa. The deviations of the mechanical characteristics’ values of the samples dealloyed at different conditions are within the standard deviation limits (Table 2). The yield strength values of the porous TiFe samples are more than two times higher when compared with the porous titanium synthesized by liquid metal dealloying from the Ti_40_Cu_60_ (at.%) precursor. Such a significant increase in strength is partially due to the alloying by β-stabilizing Fe element leading to the formation of an α + β phase structure.

## 4. Discussion

The ligament size is a common microstructural parameter used to describe the relationship between the microstructure and mechanical properties in porous metals obtained by dealloying. Porous metals with a smaller ligament size typically possess a higher strength at the nanoscale regime. This is related to the concentration of defects in these ligaments. Specifically, ligaments measuring a few nanoscales possess a significantly lower defects’ concentration when compared to their coarser counterparts. There are studies reporting the strength of ligaments approaching the theoretical strength of the material [30]. The kinetics of coarsening during dealloying can be described by an Arrhenius-type equation that is determined by diffusion coefficients. In the case of liquid metal dealloying, the process-induced coarsening is associated with the surface diffusion of elements and defines the size of the ligaments [30]. The melting point of the material can be a good indicator of its influence on the surface diffusion during liquid metal dealloying. It was found that alloying elements possessing a higher melting point led to a smaller ligament size, and vice versa, under given dealloying conditions (time and temperature). In this actual case, the melting point of the alloying element Fe (1538 °C) is lower than that of Ti (1668 °C). Therefore, a larger size of the ligaments in the porous TiFe alloy is to be expected when compared with the porous Ti synthesized by liquid metal dealloying. Moreover, it is known that the alloying of Ti by Fe enhances the sintering kinetics of titanium alloys because the mobility of titanium atoms is accelerated by the rapid diffusion of Fe [41]. Indeed, the ligaments of porous TiFe are sintered together (Figure 4c,d). However, the average size of the TiFe ligaments is comparable with that found for the porous Ti synthesized by dealloying of Ti_50_Cu_50_ (at.%) [33].

The current porous TiFe alloy samples possess an α + β-phase structure due to the alloying effect of Fe. In our previous studies, it was found that the dealloying temperature can affect the phase composition of porous Ti-based alloys [16]. However, in the current case, the same α + β-phase structure forms independently of the dealloying conditions. The observed α + β-phase structure is consistent with previous studies and was reported for other TiFe alloys with a similar Fe content [41,42].

**Figure 6:**
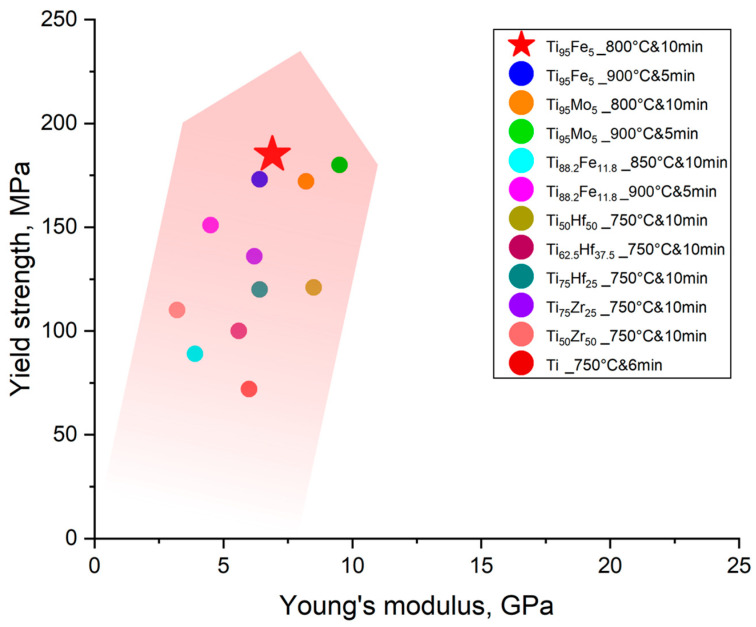
Mechanical properties of the porous titanium alloys synthesized by liquid metal dealloying based on Table 2.

The alloying by Fe is an effective approach to improve the strength of porous titanium materials obtained by liquid metal dealloying (Table 2). Among porous Ti-based alloys, the current porous TiFe alloy demonstrates one of the highest strength values (Figure 6). The relatively high strength is probably due to its α + β-phase structure as well as a relatively low fraction of pores compared to other porous Ti-based alloys obtained by liquid metal dealloying [16]. The strength can be further improved by polymer impregnation, as was shown elsewhere for porous dealloyed Ti-based alloys [16,18,19]. Despite relatively high strength values, porous TiFe demonstrates low values of Young’s modulus. These values are comparable with other open porous Ti-based alloys (Table 2) and are in the range found for human bone [43]. This suggests perspectives for the application of this material as a biomedical material.

## 5. Conclusions

This study presents a characterization of open porous TiFe alloys synthesized by liquid metal dealloying of Ti_47.5_Fe_2.5_Cu_50_ (at.%) precursor material. According to XRD analysis, the obtained TiFe alloys possess an α + β-phase structure independently of dealloying conditions, time and temperature. Mechanical tests showed that the strength of the as-dealloyed porous samples reaches about 180 MPa, while their Young’s modulus is in the range of 6–7 GPa. This demonstrates that Fe is a promising alloying element for the synthesis of porous Ti-based alloys with a moderate strength and low Young’s modulus. In turn, the combination of such favorable mechanical properties in the context of biomedical applications suggests the possibility of using the synthesized open porous TiFe alloys as implants. This requires further characterization, such as biocompatibility.

## Figures and Tables

**Table 1:** Measured composition of the porous TiFe samples. Note: the deviation in the chemical composition is likely related to the surface quality of the measured samples, namely the roughness and porosity.

Samples	Ti Fraction [at.%]	Fe Fraction [at.%]
Ti_47.5_Fe_2.5_ (800 °C and 10 min)	95.2	4.8
Ti_47.5_Fe_2.5_ (900 °C and 5 min)	94.6	5.4

**Table 2:** Mechanical properties of the porous titanium alloys synthesized by LMD.

Porous Alloy [at.%]	Phase Composition	Precursor Alloy [at.%]	T_LMD_ [°C]	t_LMD_ [min]	Yield Strength [MPa]	Young’s Modulus [GPa]	Reference
Ti_95_Fe_5_	α + β	Ti_47.5_Fe_2.5_Cu_50_	800	10	185 ± 62	6.9 ± 0.2	this study
Ti_95_Fe_5_	α + β	Ti_47.5_Fe_2.5_Cu_50_	900	5	173 ± 31	6.4 ± 2.3	this study
Ti_95_Mo_5_	α + β	Ti_47.5_Mo_2.5_Cu_50_	800	10	172 ± 28	8.2 ± 1.0	[7]
Ti_95_Mo_5_	α + β	Ti_47.5_Mo_2.5_Cu_50_	900	5	180 ± 66	9.5 ± 1.1	[7]
Ti_88.2_Fe_11.8_	β	Ti_29.2_Fe_3.9_Cu_66.9_	850	10	89 ± 10	3.9 ± 0.3	[16]
Ti_88.2_Fe_11.8_	β	Ti_29.2_Fe_3.9_Cu_66.9_	900	5	151 ± 12	4.5 ± 0.4	[16]
Ti	α	Ti_40_Cu_60_	750	6	72 ± 5	6.0 ± 0.3	[18]
Ti_50_Hf_50_	α	Ti_20_Hf_20_Cu_60_	750	10	121 ± 12	8.5 ± 1.3	[19]
Ti_62.5_Hf_37.5_	α	Ti_25_Hf_15_Cu_60_	750	10	100 ± 8	5.6 ± 1.0	[19]
Ti_75_Hf_25_	α	Ti_30_Hf_10_Cu_60_	750	10	120 ± 6	6.4 ± 0.5	[19]
Ti_75_Zr_25_	α	Ti_30_Zr_10_Cu_60_	750	10	136 ± 10	6.2 ± 0.7	[15]
Ti_50_Zr_50_	α	Ti_15_Zr_15_Cu_70_	750	10	110 ± 10	3.2 ± 0.2	[15]

## Data Availability

Data will be made available on request.

## References

[1] Kujala S., Ryhänen J., Danilov A., Tuukkanen J. (2003). Effect of porosity on the osteointegration and bone ingrowth of a weight-bearing nickel–titanium bone graft substitute. Biomaterials.

[2] Lewallen E.A., Riester S.M., Bonin C.A., Kremers H.M., Dudakovic A., Kakar S., Cohen R.C. (2015). Biological Strategies for Improved Osseointegration and Osteoinduction of Porous Metal Orthopedic Implants. Tissue Eng. Part B Rev..

[3] Niinomi M., Nakai M., Hieda J. (2012). Development of new metallic alloys for biomedical applications. Acta Biomater..

[4] Niinomi M. (2008). Biologically and Mechanically Biocompatible Titanium Alloys. Mater. Trans..

[5] Thoemmes A., Ivanov I.V., Ruktuev A.A., Lazurenko D.V., Bataev I.A. (2019). Structure and Phase Composition of Biomedical Alloys of the Ti–Nb System in Cast Condition and After Heat Treatment. Met. Sci. Heat Treat..

[6] Geetha M., Singh A.K., Asokamani R., Gogia A.K. (2009). Ti based biomaterials, the ultimate choice for orthopaedic implants—A review. Prog. Mater. Sci..

[7] Berger S.A., Okulov I.V. (2020). Open porous α + β titanium alloy by liquid metal dealloying for biomedical applications. Metals.

[8] Nakai M., Niinomi M., Akahori T., Tsutsumi H., Itsuno S., Haraguchi N., Itoh Y., Ogasawara T. (2010). Development of biomedical porous titanium filled with medical polymer by in-situ polymerization of monomer solution infiltrated into pores. J. Mech. Behav. Biomed. Mater..

[9] Zhang L.-C., Chen L.-Y. (2019). A Review on Biomedical Titanium Alloys: Recent Progress and Prospect. Adv. Eng. Mater..

[10] Van der Stok J., Van der Jagt O.P., Amin Yavari S., De Haas M.F.P., Waarsing J.H., Jahr H., Van Lieshout E.M.M., Patka P., Verhaar J.A.N., Zadpoor A.A. (2013). Selective laser melting-produced porous titanium scaffolds regenerate bone in critical size cortical bone defects. J. Orthop. Res..

[11] Putra N.E., Mirzaali M.J., Apachitei I., Zhou J., Zadpoor A.A. (2020). Multi-material additive manufacturing technologies for Ti-, Mg-, and Fe-based biomaterials for bone substitution. Acta Biomater..

[12] Shalnova S.A., Kuzminova Y.O., Evlashin S.A., Klimova-Korsmik O.G., Vildanov A.M., Shibalova A.A., Turichin G.A. (2022). Effect of recycled powder content on the structure and mechanical properties of Ti-6Al-4V alloy produced by direct energy deposition. J. Alloys Compd..

[13] Tomoyuki F., Ryo M., Naoto K., Keiichiro T., Yoshinobu S. (2022). Uniform porous and functionally graded porous titanium fabricated via space holder technique with spark plasma sintering for biomedical applications. Adv. Powder Technol..

[14] Okulov A.V., Iusupova O.S., Kazantseva N.V. (2023). Liquid metal dealloying combined with polymer impregnation as novel promising technology for bioHEA-based implant manufacturing. E3S Web Conf..

[15] Okulov I.V., Okulov A.V., Soldatov I.V., Luthringer B., Willumeit-Römer R., Wada T., Kato H., Weissmüller J., Markmann J. (2018). Open porous dealloying-based biomaterials as a novel biomaterial platform. Mater. Sci. Eng. C.

[16] Okulov I.V., Okulov A.V., Volegov A.S., Markmann J. (2018). Tuning microstructure and mechanical properties of open porous TiNb and TiFe alloys by optimization of dealloying parameters. Scr. Mater..

[17] Nakai M., Niinomi M., Ishii D. (2011). Mechanical and biodegradable properties of porous titanium filled with poly-L-lactic acid by modified in situ polymerization technique. J. Mech. Behav. Biomed. Mater..

[18] Okulov I.V., Weissmüller J., Markmann J. (2017). Dealloying-based interpenetrating-phase nanocomposites matching the elastic behavior of human bone. Sci. Rep..

[19] Okulov A.V., Volegov A.S., Weissmüller J., Markmann J., Okulov I.V. (2018). Dealloying-based metal-polymer composites for biomedical applications. Scr. Mater..

[20] Wada T., Yubuta K., Inoue A., Kato H. (2011). Dealloying by metallic melt. Mater. Lett..

[21] Geslin P., Mccue I., Erlebacher J., Karma A. (2015). Topology-generating interfacial pattern formation during liquid metal dealloying. Nat. Commun..

[22] McCue I., Gaskey B., Geslin P.A., Karma A., Erlebacher J. (2016). Kinetics and morphological evolution of liquid metal dealloying. Acta Mater..

[23] Xiang Y.-H., Liu L.-Z., Shao J.-C., Jin H.-J. (2020). A universal scaling relationship between the strength and Young’s modulus of dealloyed porous Fe_0.80_Cr_0.20_. Acta Mater..

[24] Joo S.-H., Yubuta K., Kato H. (2020). Ordering kinetics of nanoporous FeCo during liquid metal dealloying and the development of nanofacets. Scr. Mater..

[25] Park W.-Y., Wada T., Joo S.-H., Han J., Kato H. (2020). Novel hierarchical nanoporous graphene nanoplatelets with excellent rate capabilities produced via self-templating liquid metal dealloying. Mater. Today Commun..

[26] Mokhtari M., Bourlot C.L., Adrien J., Bonnin A., Wada T., Duchet-Rumeau J., Kato H., Maire E. (2018). Microstructure characterization by X-ray tomography and EBSD of porous FeCr produced by liquid metal dealloying. Mater. Charact..

[27] Okulov I.V., Joo S.-H., Okulov A.V., Volegov A.S., Luthringer B., Willumeit-Römer R., Zhang L., Mädler L., Eckert J., Kato H. (2020). Surface functionalization of biomedical Ti-6Al-7Nb alloy by liquid metal dealloying. Nanomaterials.

[28] McCue I., Ryan S., Hemker K., Xu X., Li N., Chen M., Erlebacher J. (2016). Size Effects in the Mechanical Properties of Bulk Bicontinuous Ta/Cu Nanocomposites Made by Liquid Metal Dealloying. Adv. Eng. Mater..

[29] Okulov A.V., Joo S.-H., Kim H.S., Kato H., Okulov I.V. (2020). Nanoporous high-entropy alloy by liquid metal dealloying. Metals.

[30] Joo S.-H., Bae J.W., Park W.-Y., Shimada Y., Wada T., Kim H.S., Takeuchi A., Konno T.J., Kato H., Okulov I.V. (2020). Beating Thermal Coarsening in Nanoporous Materials via High-Entropy Design. Adv. Mater..

[31] Okulov I.V., Geslin P.-A., Soldatov I.V., Ovri H., Joo S.-H., Kato H. (2019). Anomalously low modulus of the interpenetrating-phase composite of Fe and Mg obtained by liquid metal dealloying. Scr. Mater..

[32] Okulov I.V., Lamaka S.V., Wada T., Yubuta K., Zheludkevich M.L., Weissmüller J., Markmann J., Kato H. (2018). Nanoporous magnesium. Nano Res..

[33] Tsuda M., Wada T., Kato H. (2013). Kinetics of formation and coarsening of nanoporous α-titanium dealloyed with Mg melt. J. Appl. Phys..

[34] Wada T., Setyawan A.D., Yubuta K., Kato H. (2011). Nano- to submicro-porous β-Ti alloy prepared from dealloying in a metallic melt. Scr. Mater..

[35] Leyens C., Peters M. (2003). Titanium and Titanium Alloys.

[36] Ilyin A.A., Kolachev B.A., Polkin I.S. (2009). Titanium Alloys. Composition, Structure, Properties.

[37] Takeuchi A., Inoue A. (2005). Metallic Glasses By Atomic Size Difference, Heat of Mixing and Period of Constituent Elements and Its Application To Characterization of the Main Alloying Element. Mater. Trans..

[38] Straumal B.B., Kilmametov A.R., Ivanisenko Y., Gornakova A.S., Mazilkin A.A., Kriegel M.J., Fabrichnaya O.B., Baretzky B., Hahn H. (2015). Phase Transformations in Ti–Fe Alloys Induced by High-Pressure Torsion. Adv. Eng. Mater..

[39] Straumal B.B., Kilmametov A.R., Mazilkin A.A., Gornakova A.S., Fabrichnaya O.B., Kriegel M.J., Rafaja D., Bulatov M.F., Nekrasov A.N., Baretzky B. (2020). Formation of the ω Phase in the Titanium–Iron System under Shear Deformation. JETP Lett..

[40] Wei W., Liu Y., Zhou K., Huang B. (2003). Effect of Fe addition on sintering behaviour of titanium powder. Powder Metall..

[41] Zadorozhnyy V.Y., Inoue A., Louzguine-Luzgin D.V. (2012). Ti-based nanostructured low-alloy with high strength and ductility. Mater. Sci. Eng. A.

[42] Romero C., Yang F., Wei S., Bolzoni L. (2020). Thermomechanical Processing of Cost-Affordable Powder Metallurgy Ti-5Fe Alloys from the Blended Elemental Approach: Microstructure, Tensile Deformation Behavior, and Failure. Metals.

[43] Smallman R.E., Bishop R.J., Smallman R.E., Bishop R.J. (1999). Chapter 13–Biomaterials. Modern Physical Metallurgy and Materials Engineering.

